# Comparative Analysis of Three-Dimensional Chromatin Architecture in the Shell Gland Between Blue- and White-Shelled Laying Ducks

**DOI:** 10.3390/biology15211957

**Published:** 2026-11-01

**Authors:** Yingping Wu, Jindong Ren, Lizhi Lu, Li Chen, Xiaoyu Zhao

**Affiliations:** 1 College of Animal Science, Xinjiang Agricultural University, Urumqi 830052, China; 2 Institute of Animal Husbandry and Veterinary Science, Xianghu Laboratory, Zhejiang Academy of Agricultural Sciences, Hangzhou 311231, China

**Keywords:** three-dimensional genome, laying duck, shell gland, chromatin architecture, eggshell color

## Abstract

Chromosomal three-dimensional (3D) structural variations can mediate phenotypic differentiation in animals and plants by altering chromatin interaction patterns and regulating gene transcription. Eggshell color is an important economic trait of laying ducks, and the uterus (shell gland) of the oviduct is the core functional site for eggshell formation and pigment deposition. To reveal the 3D genomic regulatory mechanism underlying eggshell color differentiation in blue-shelled laying ducks, this study employed high-throughput chromosome conformation capture (Hi-C) technology to systematically compare chromosomal spatial conformation differences in the shell glands between blue-shelled and white-shelled laying ducks and to dissect the regulatory characteristics of chromatin architecture at different hierarchical levels. The results showed that both phenotypic groups possessed typical and conserved chromatin interaction patterns; however, their spatial folding characteristics differed significantly. Blue-shelled laying ducks (Q1) exhibited predominantly short-range loose chromatin interactions, whereas white-shelled laying ducks (B1) tended to have medium- and long-range large-scale DNA looping interactions. A/B compartment analysis revealed that chromosomal compartmentalization differed little between the two groups, with dynamic transitions occurring in both A and B compartments. Differentially compartmentalized genes were mainly enriched in pathways such as immune response, basal metabolism, reproductive development, calcium signaling, and estrogen signaling. TAD topological structure analysis revealed obvious TAD boundary remodeling on chromosome 4 in blue-shelled laying ducks and significant differences in TAD number, length, and boundary signal strength between the two groups. Changes in specific TAD boundaries also led to altered gene functions. Differentially expressed genes were mainly enriched in processes such as cell–cell adhesion, metabolism, and cell cycle, as well as regulatory pathways closely related to pigment deposition, including porphyrin and chlorophyll metabolism, melanin biosynthesis, riboflavin metabolism, and bile secretion. In summary, based on chromatin spatial conformation analysis, this study found that the eggshell color trait of laying ducks did not appear to depend primarily on macroscopic A/B compartment structural regulation; however, it was primarily associated with local specific TAD topological remodeling. This remodeling may be linked to gene expression in pigment deposition-related pathways by altering local chromatin interaction patterns. Given the limited sample size, these findings are preliminary and suggest a potential association between 3D chromatin architecture and eggshell color, which warrants further functional validation.

## Simple Summary

Eggshell color is an important economic trait for laying ducks. This study used Hi-C technology to compare the three-dimensional structure of chromatin in the shell glands of blue-shelled and white-shelled laying ducks. The overall interaction patterns of the two groups are conservative, with blue-shelled laying ducks mainly exhibiting close-range loose interactions, while white-shelled laying ducks tend to exhibit chromatin circularization at medium to long distances. The A/B compartments are highly stable, while specific TAD boundary remodeling occurs on chromosome 4 of blue-shelled laying ducks and is related to the regulation of pigment deposition genes. Local TAD topological variation is the key to eggshell color differentiation, and this study provides a basis for molecular marker-assisted breeding to elucidate differences in eggshell color in ducks.

## 1. Introduction

Chromosomes are the largest organizational units in eukaryotic genomes. Genomic DNA is stored in the nucleus through highly folded and condensed chromatin, forming a relatively complex three-dimensional (3D) chromatin structure. Chromosome folding occurs hierarchically and progressively, generating conformations such as chromatin loops, topologically associating domains (TADs), transcriptionally active and silent A/B compartments, and chromosome territories across scales from small to large [1]. These structures exhibit distinct characteristics at different scales [2,3]. Studies have shown that changes in 3D genome architecture and chromosomal interaction strength can affect gene expression, thereby leading to phenotypic variation [4]. Consequently, 3D genomics, which aims to explain the regulatory mechanisms of gene expression based on chromatin spatial conformation, has emerged. Currently, the high-throughput chromosome conformation capture (Hi-C) technique proposed by Lieberman-Aiden et al. [5] in 2009 has been widely applied, enabling the capture and detection of interactions between chromosomal fragments across the entire genome.

An increasing number of studies have revealed that 3D genome architecture plays important roles in gene expression and cellular function regulation. For example, Shao et al. [6] used Hi-C technology to compare the 3D genome architecture of hepatocytes from Lindian chickens (LDC) and Wenchang chickens (WCC) and found that environmental adaptation contributed to A/B compartment switching and reorganization of TADs and TAD boundaries in the two types of hepatocytes. Fishman performed genome-wide analysis of chromatin interactions in chicken embryonic fibroblasts (CEFs) and adult erythrocytes. They found that chicken erythrocytes differed greatly from fibroblasts, displaying obvious A and B compartments, lacking typical TADs, and forming long-range chromatin interactions previously observed on mitotic chromosomes [7]. Maslova et al. [8] analyzed the contact patterns of Hi-C maps from the chicken LSCC-HD3 cell line (HD3) derived from erythroid progenitors and identified interchromosomal translocations in regions ≥200 kb, which mainly occurred on microchromosomes and macrochromosomes.

The reproductive organs of female ducks consist of a developed left ovary and oviduct [9]. The oviduct includes the infundibulum, magnum, isthmus, uterus, and vagina. During egg formation, the egg remains in the uterus for the longest time, and this is also the site of eggshell formation and pigment deposition [10]. Eggshell color is one of the reasons consumers choose poultry eggs [11]. Eggshell formation is a highly complex process and is precisely controlled by genetic and biological pathways in the uterus [12,13,14,15]. Therefore, changes in uterine function may significantly affect eggshell quality and eggshell pigmentation [16]. However, differences in the 3D chromosomal architecture of the uterine genome among laying ducks with different eggshell colors have not been reported. Therefore, in this study, we analyzed the 3D chromatin architecture of the uterus in laying ducks with different eggshell colors using Hi-C technology, aiming to provide clues for further understanding the regulatory mechanism of the blue-shelled gene.

## 2. Materials and Methods

### 2.1. Sample Collection

White-shelled laying ducks (homozygous recessive genotype) and blue-shelled laying ducks (homozygous dominant genotype) at the laying stage with stable egg production were selected. After anesthesia, the ducks were sacrificed by exsanguination via the jugular vein. The abdominal cavity was opened, and the shell gland was collected. Samples were labeled as B1 (white-shelled laying ducks) and Q1 (blue-shelled laying ducks), with two biological replicates per group. All samples were immediately snap-frozen in liquid nitrogen and subsequently stored at −80 °C for Hi-C library construction and sequencing.

### 2.2. Hi-C Library Construction and Sequencing

Shell gland samples were cross-linked with 1% formaldehyde (Sigma-Aldrich, St. Louis, MO, USA) at room temperature for 10 min, and the cross-linking reaction was quenched with glycine at a final concentration of 0.125 M for 5 min. Cells were lysed, and endogenous nucleases were inactivated with 0.3% SDS (Sigma-Aldrich, St. Louis, MO, USA). Chromatin DNA was then digested with 100 U MboI (NEB, Ipswich, MA, USA) and labeled with biotin-14-dCTP (Invitrogen, Carlsbad, CA, USA). Subsequently, ligation was performed with 50 U T4 DNA ligase (NEB, Ipswich, MA, USA). After de-cross-linking, the ligated DNA was extracted using a QIAamp DNA Mini Kit (Qiagen, Hilden, Germany) according to the manufacturer’s instructions. The purified DNA was sheared into 300–500 bp fragments, followed by end repair, A-tailing, and adapter ligation. DNA fragments were then pulled down with biotin–streptavidin magnetic beads(Dynabeads™ MyOne™ Streptavidin C1; Invitrogen, Carlsbad, CA, USA) and amplified using PCR. Finally, the Hi-C libraries were quantified, and qualified libraries were sequenced on an Illumina Nova-seq platform (San Diego, CA, USA) or an MGI-seq platform (MGI, Shenzhen, China).

### 2.3. Hi-C Data Processing and Normalization

First, raw sequencing reads were filtered, and quality assessment was performed using Trimmomatic(version 0.39) [17] and FastQC (version 0.11.9) software with default parameters [18] to generate clean reads. The clean reads were mapped to the mallard duck genome (Assembly: GCA_008746955.1). As the wild ancestor of domestic laying ducks, mallard shares the same species with domestic ducks, and this assembly is the latest and most complete reference for this species. Valid reads were retrieved using the hiclib pipeline after genome mapping. Intra- and interchromosomal interactions at 100 kb resolution were selected using Fit-Hi-C (version 2.0.7) software [19]. To visualize differences in chromatin interaction patterns between the two groups, a differential interaction matrix was generated following the method of Crane et al. [20]. Specifically, for each group, the two biological replicates were first pooled to generate a group-level interaction matrix. Each group-level matrix was then converted into a Z-score matrix, and the Z-score matrix of one group was subtracted from that of the other to obtain a differential interaction matrix. This matrix subtraction was used solely for visual and descriptive comparison of interaction patterns.

### 2.4. Identification of A/B Compartments

Chromosomal compartments are highly plastic and undergo A/B compartment switching under different cell types and biological states, leading to large-scale changes in gene expression activity. We performed comparative analysis of compartments among different samples at 100 kb resolution. CscoreTool (version 1.1) was used for normalization to identify A and B compartments at 100 kb resolution. For each chromosome, genomic regions with positive c-score signals were classified as compartment A, and those with negative c-score signals were classified as compartment B [21].

### 2.5. Identification of TADs

TADs (topologically associating domains) are the basic organizational units of the genome in spatial structure. We performed comparative analysis of TADs among different samples at 20 kb resolution. Based on the normalized intrachromosomal interaction matrices at 20 kb resolution, TADs were identified by insulation score (IS) following the method described in a previous study [22].

### 2.6. Distribution of Relative Signal Values of TAD Boundaries

According to the method reported in the literature [22], the dynamic changes in TAD structure can be investigated by calculating the relative signal values of TAD boundaries. Shared TAD boundaries between samples that were not located within 2 Mb from the ends of chromosomes were selected, and boundaries with fewer than 10 interactions within 2 Mb upstream and downstream were filtered out. The ratio of interactions at the boundary to those within 2 Mb upstream and downstream was calculated and log2-transformed to obtain the signal value of the TAD boundary. The minimum value was then subtracted from all values, finally yielding the distribution of relative signal values of TAD boundaries for each sample.

### 2.7. Enrichment Analysis

KEGG pathway enrichment analysis of genes within differential regions and genes residing inside specific TAD boundaries was performed using KOBAS 2.0 [23]. This analysis identifies significantly enriched pathways by applying the hypergeometric test based on the KEGG database (https://www.kegg.jp/, accessed on 10 October 2024) [24]. Gene Ontology (GO) enrichment analysis was conducted with GOseq(version 1.44.0) [25], which implements the Wallenius non-central hypergeometric distribution to account for selection bias in sequencing data. Pathways and GO terms with *p* < 0.05 were considered significantly enriched.

## 3. Results

### 3.1. Hi-C Sequencing Data Description

Hi-C sequencing was performed on shell glands from white-shelled laying ducks (B1) and blue-shelled laying ducks (Q1), with two individuals per group. A total of 1,787,846,376 and 1,749,723,531 clean reads were obtained for B1 and Q1, respectively. Q20 was above 98.1%, Q30 was above 93.8%, and GC content ranged from 42.4% to 43.4% (Table 1), indicating that the Hi-C sequencing data were of high quality and met the requirements for subsequent analyses.

As shown in Table 2, the valid pairs of the four samples ranged from 58.62% to 65.19%. The proportions of intrachromosomal (cis) and interchromosomal (trans) interactions were further calculated. The average cis/trans ratio was 2.65 in the B1 group and 3.35 in the Q1 group. The results indicated that intrachromosomal cis interactions predominated in both genotypes and that the proportion of intrachromosomal interactions in the Q1 group was higher than that in the B1 group.

### 3.2. Comparative Analysis of Genome-Wide Interaction Maps in the Shell Glands of Blue- and White-Shelled Laying Ducks

**Figure 1:**
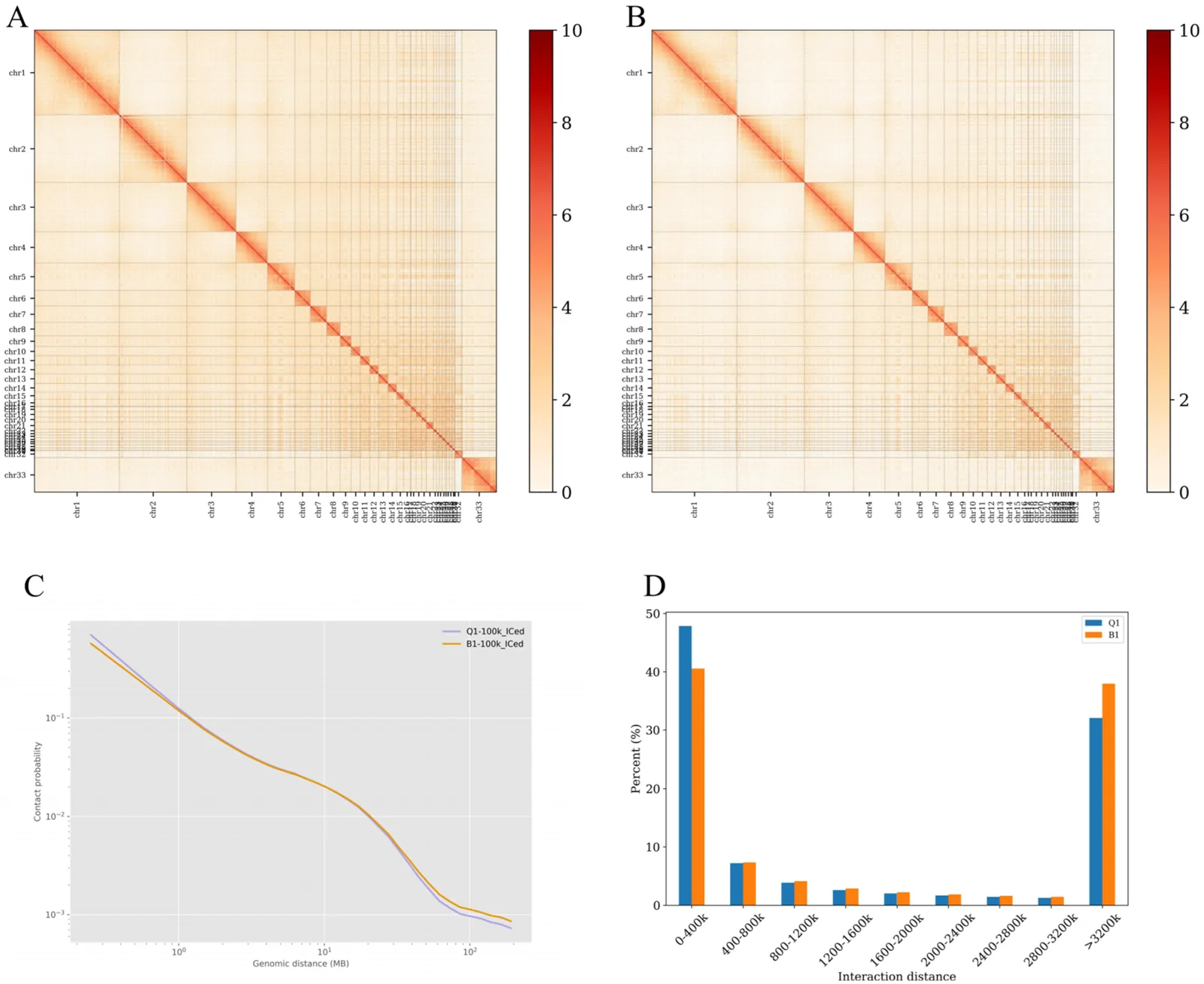
Genome-wide interaction maps of the shell glands from blue-shelled and white-shelled laying ducks. (**A**) Heatmap of the genome-wide interaction matrix at 100 kb resolution for B1; (**B**) Heatmap of the genome-wide interaction matrix at 100 kb resolution for Q1; (**C**) Genome-wide distance–interaction frequency plot at 20 kb resolution; (**D**) Distribution of interaction proportions across different distance ranges. Note: The dense labels of microchromosomes (chr24–chr33) in panels A and B are due to the whole-genome scale and do not affect the visualization of macroscopic patterns.

Chromatin interaction heatmaps (Figure 1A,B) showed that both Q1 and B1 exhibited extensive interactions across the entire genome (100 kb bins). Both groups displayed strong diagonal interaction signals, and intrachromosomal interaction strength was greater than interchromosomal interaction strength, indicating tight associations and high-frequency interactions between loci at adjacent positions on chromosomes. Overall, however, the interaction strength in the B1 group was greater than that in the Q1 group, further indicating that the chromosomal structure in the Q1 group was relatively looser. Using comparative analysis of the differences in distance and interaction frequency between samples (Figure 1C), it was observed that the genome-wide interaction decay trends were generally similar between Q1 and B1, with interaction frequency decreasing as interaction distance increased in both groups. In the 0-1 Mb range, the interaction frequency in the Q1 group was greater than that in the B1 group, whereas in the 10-100 Mb range, the interaction frequency in the B1 group was greater than that in the Q1 group. This reflected that intrachromosomal interactions in the B1 group were more inclined toward medium- and long-range interactions, forming larger DNA loop structures, while short-range interaction frequencies were relatively lower.

### 3.3. Comparative Analysis of Compartments in the Shell Glands of Blue- and White-Shelled Laying Ducks

#### 3.3.1. Genome-Wide Landscape of A/B Compartments

**Figure 2:**
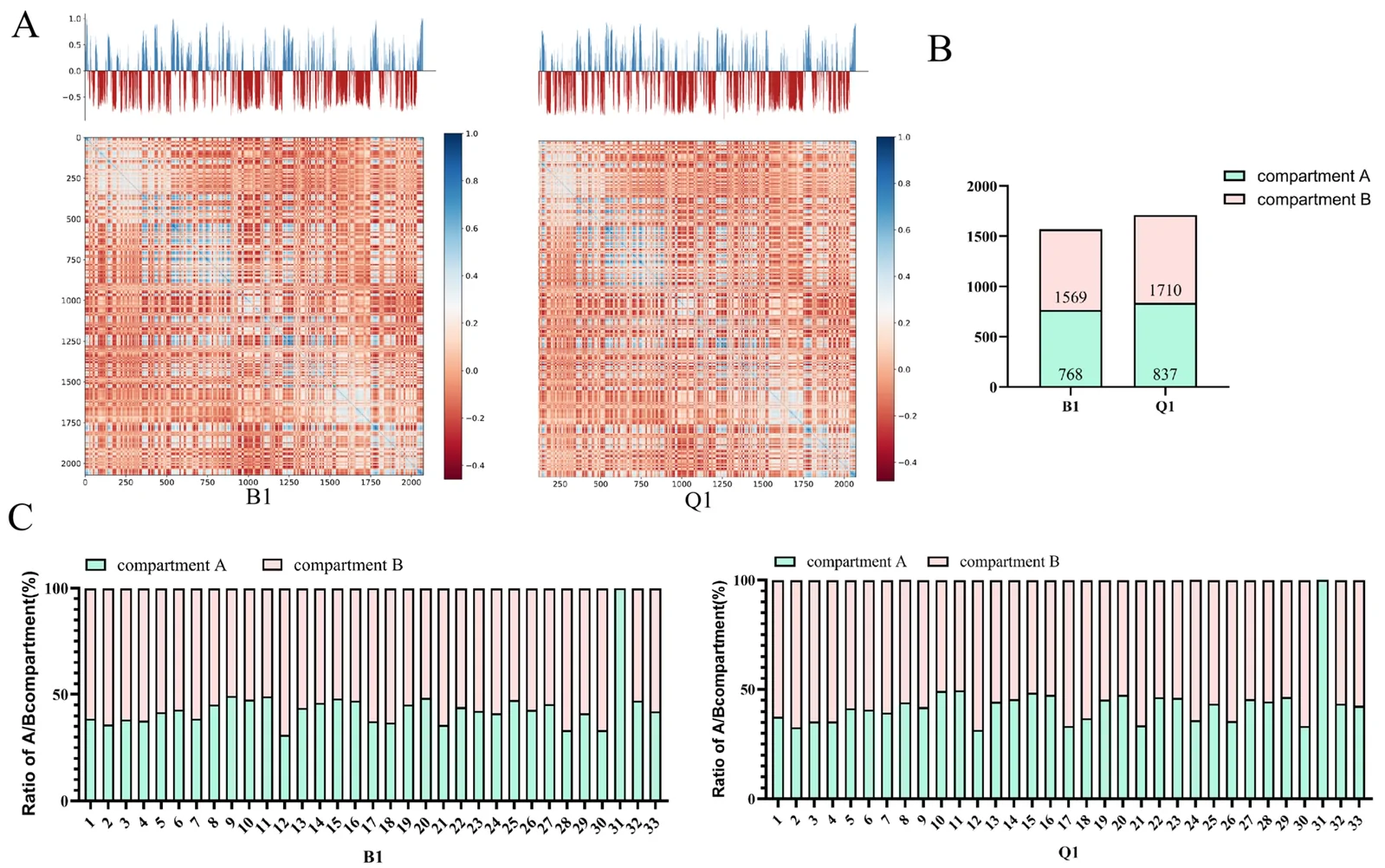
Identification of A/B compartments. (**A**) Representative compartment profile using chromosome 1 as an example; (**B**) Distribution of A/B compartments in B1 and Q1; (**C**) Proportion of A/B compartments on each chromosome.

The results showed that intrachromosomal interactions in duck shell glands exhibited a typical checkerboard distribution pattern, indicating the presence of local interaction-related structures within chromosomes (Figure 2A). The A/B c-score distributions of the B1 and Q1 groups were mainly concentrated between −0.9999 and 0.9999, representing a relatively typical normal distribution. In the two groups, the proportions of compartment A and compartment B on each chromosome were calculated. The proportions of compartments A/B were identical between the two groups. Specifically, compartment A accounted for 48.9% and compartment B accounted for 51.1% in both groups, with compartment B occupying a higher proportion of the genome than compartment A (Figure 2B, Table S1). Notably, the A/B proportions varied among different chromosomes in both groups (Figure 2C). Chromosome 31 was entirely compartment A in individuals of both genotypes, indicating that chromatin compartments varied considerably among individual chromosomes.

#### 3.3.2. Functional Annotation of Genes in Differential Compartment Regions

**Figure 3:**
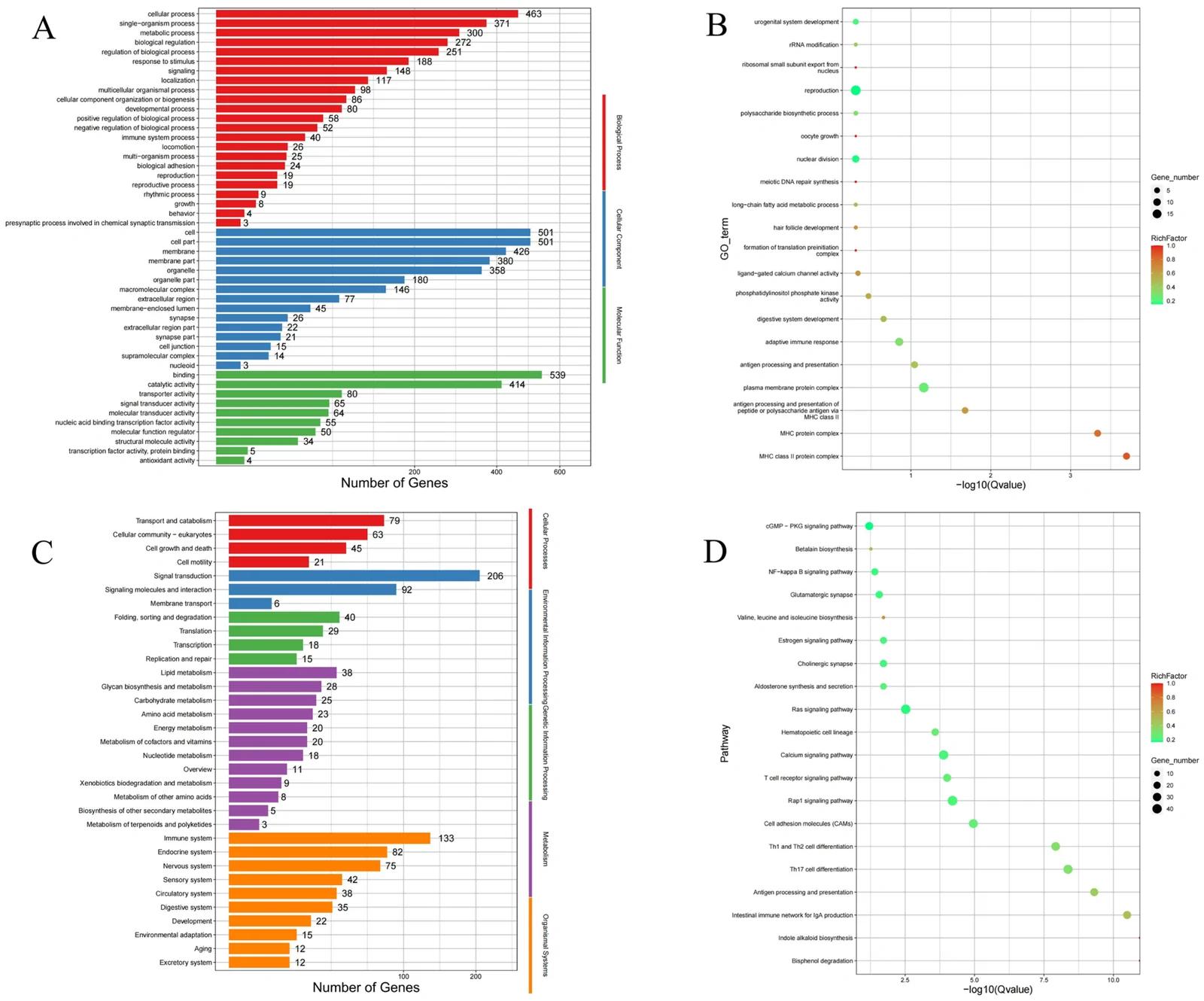
Significant GO and KEGG enrichment results for genes within differential compartments between samples. (**A**) GO enrichment classification of genes within differential compartment regions between pairwise samples; (**B**) GO enrichment results of genes within differential compartment regions between pairwise samples; (**C**) KEGG enrichment classification of genes within differential compartment regions between pairwise samples; (**D**) KEGG enrichment results of genes within differential compartment regions between pairwise samples.

To investigate the functions of genes within differential compartments between blue-shelled and white-shelled laying ducks samples, GO functional annotation was performed on genes located in differential compartments. The results showed that 292 of 4146 GO terms were significantly enriched (*p* < 0.05) (Figure 3A,B). Genes involved in biological processes (67.8%) were significantly enriched in antigen processing and presentation of peptide or polysaccharide antigen via MHC class II, reproduction, and oocyte growth. Genes involved in cellular components (11.6%) were significantly enriched in the MHC protein complex, plasma membrane protein complex, and Golgi membrane. Genes involved in molecular functions (20.5%) were significantly enriched in phosphatidylinositol phosphate kinase activity, ligand-gated calcium channel activity, and nucleotide binding.

A total of 243 KEGG pathways were enriched for genes within differential compartments between samples (Figure 3C,D), with 44 pathways significantly enriched (*p* < 0.05), including indole alkaloid biosynthesis, bisphenol degradation, antigen processing and presentation, calcium signaling pathway, and estrogen signaling pathway. Neither GO nor KEGG analysis revealed GO terms or pathways related to eggshell color formation, indicating that chromosomal compartment differences were not involved in eggshell color formation.

### 3.4. Comparative Analysis at the TAD Level in the Shell Glands of Blue- and White-Shelled Laying Ducks

**Figure 4:**
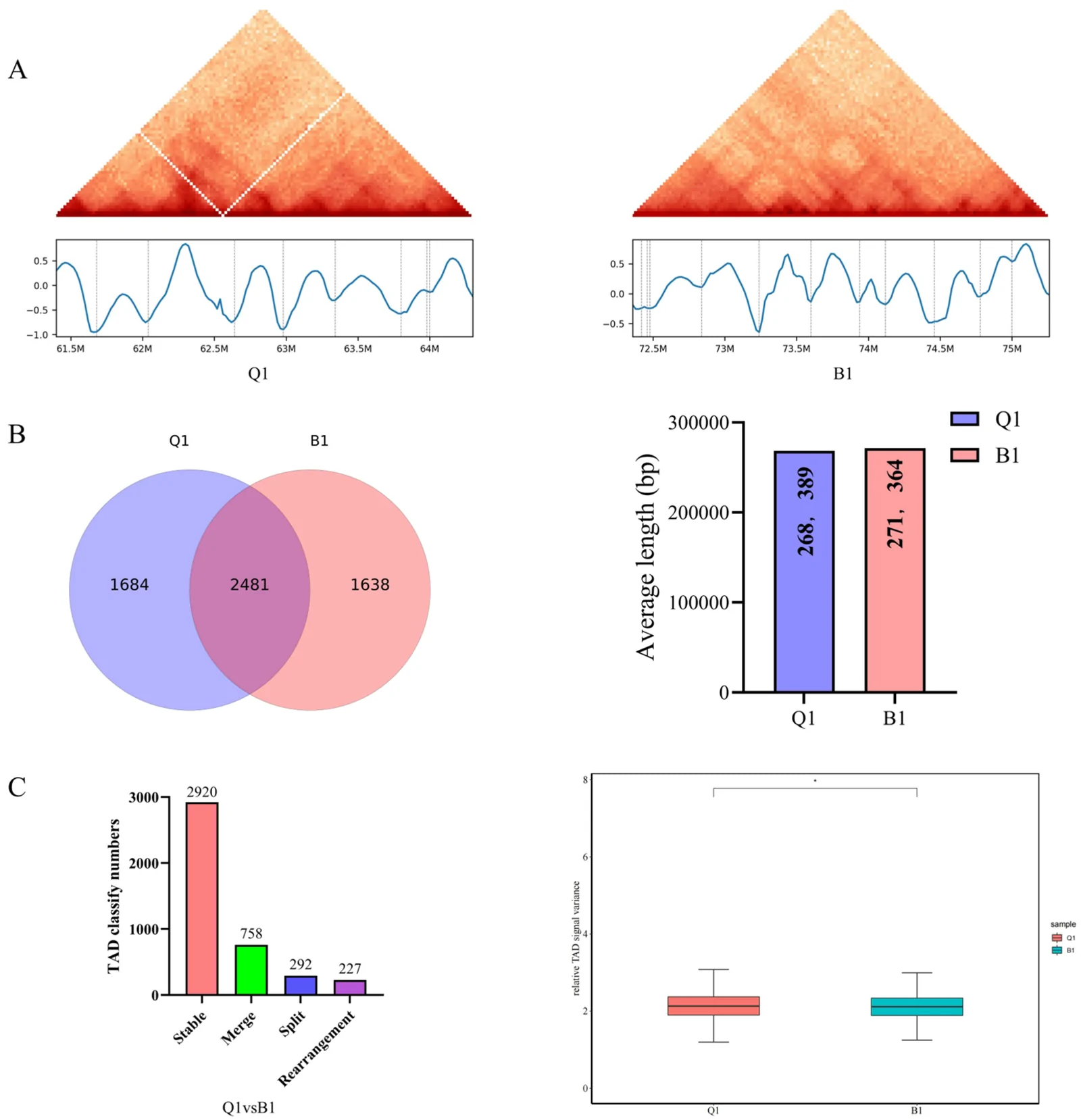
Comparative analysis of TAD structures in the shell glands of blue-shelled and white-shelled laying ducks. (**A**) TAD display on chromosome 4, the intersecting white lines in the Q1 heatmap highlight the specific genomic region where TAD boundary repositioning or disappearance occurred; (**B**) Number and length of TAD boundaries between samples; (**C**) Classification of TAD comparisons between samples and distribution of relative signal values of TAD boundaries between samples. * indicates *p* < 0.05.

This study analyzed and compared the characteristics of topologically associating domains (TADs) in the shell glands of blue-shelled laying ducks (Q1) and white-shelled laying ducks (B1) (Figure 4). The results showed that TADs differed between B1 and Q1 tissues in the same chromosomal regions. It could be observed that the TAD structure of the Q1 group on chromosome 4 (chr4) underwent obvious changes in some regions (Figure 4A), manifesting as the repositioning or disappearance of TAD boundaries, which might affect chromatin interactions and expression regulation of the genes within them. The insulation score curve further indicated that TAD boundaries differed between B1 and Q1. The number and length of TAD boundaries between samples at 20 kb resolution were calculated (Figure 4B). As shown in the figure, the Q1 group had 4165 TAD boundaries with an average length of 268,389 bp, while the B1 group had 4119 TAD boundaries with an average length of 271,364 bp; a total of 2481 TAD boundaries were shared between the two groups. These results indicated that the B1 group had fewer TADs and longer TADs than the Q1 group. The distribution of relative signal values of TAD boundaries for each sample was obtained with calculation (Figure 4C). The distribution of relative signal values of TAD boundaries between B1 and Q1 was Q1 > B1 (*p* < 0.05) (Figure 4C, right). As shown in the figure (Figure 4C, left), compared with B1, the predominant TAD type in Q1 was Stable (69.6%), followed by Merge (18.1%), Split (7.0%), and Rearrangement (5.4%).

**Figure 5:**
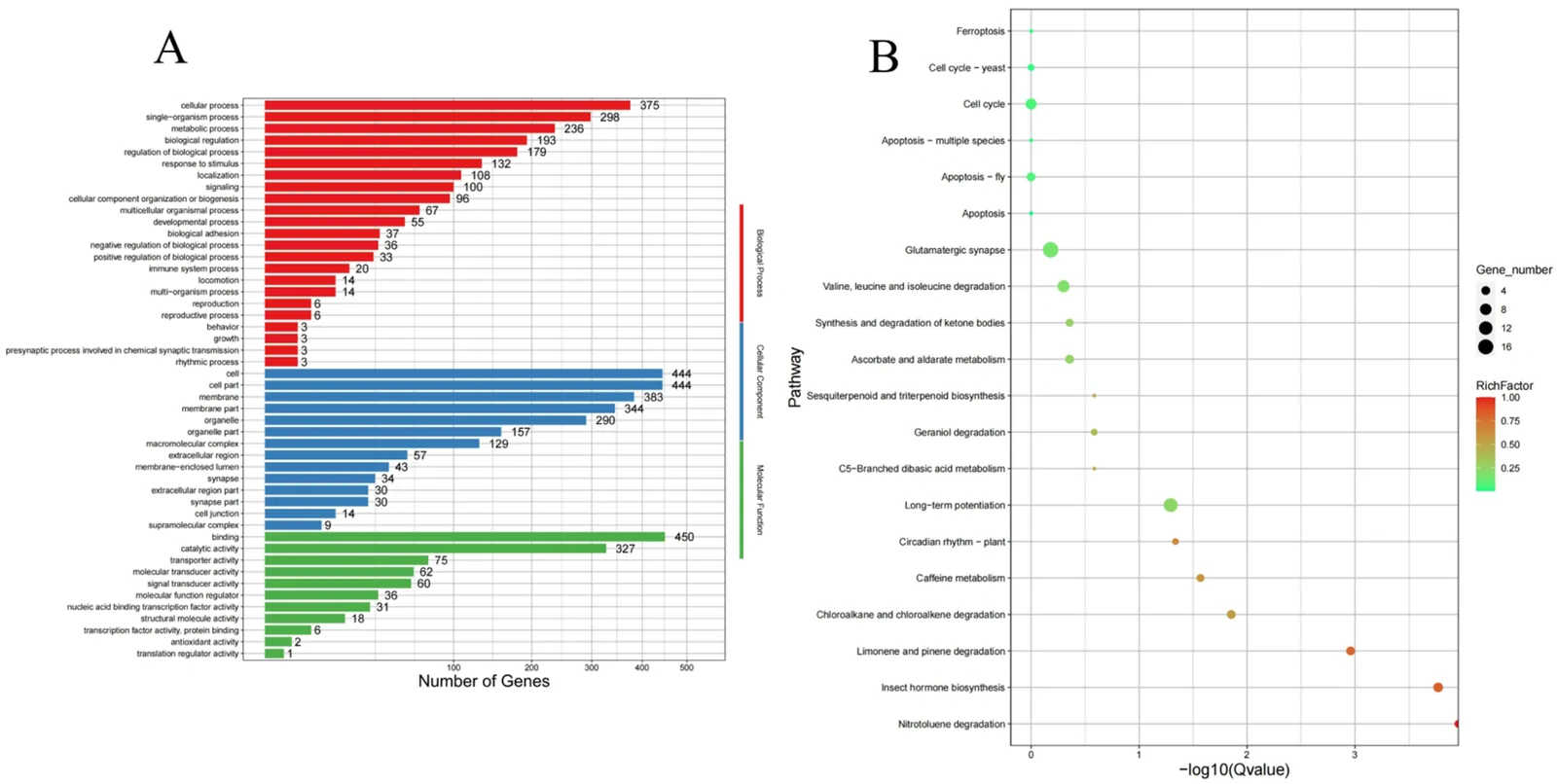
GO and KEGG enrichment analysis of differential TAD boundaries in the B1 group. (**A**) Bar plot of GO enrichment for differential TAD boundaries in the B1 group; (**B**) Bubble plot of KEGG enrichment for differential TAD boundaries in the B1 group.

Changes in TAD boundaries can affect gene expression. Therefore, this study investigated the functions of genes located within specific TAD boundaries. Functional annotation was performed on gene sets located within differential TAD boundaries (Tables S2–S5). In the B1 group, 179 of 3289 GO terms were significantly enriched (*p* < 0.05) (Figure 5A). Genes involved in biological processes (62.7%) were significantly enriched in homophilic cell adhesion via plasma membrane adhesion molecules, cell–cell adhesion, and galactose metabolic process. Genes involved in cellular components (13.8%) were significantly enriched in synapse part, ion channel complex, and neuron part. Genes involved in molecular functions (23.3%) were significantly enriched in galactokinase activity, ion channel activity, and transmitter-gated ion channel activity. KEGG enrichment analysis of the B1 group (Figure 5B) showed that a total of 228 KEGG pathways were enriched, among which 14 pathways were significantly enriched (*p* < 0.05), including nitrotoluene degradation, insect hormone biosynthesis, and limonene and pinene degradation.

**Figure 6:**
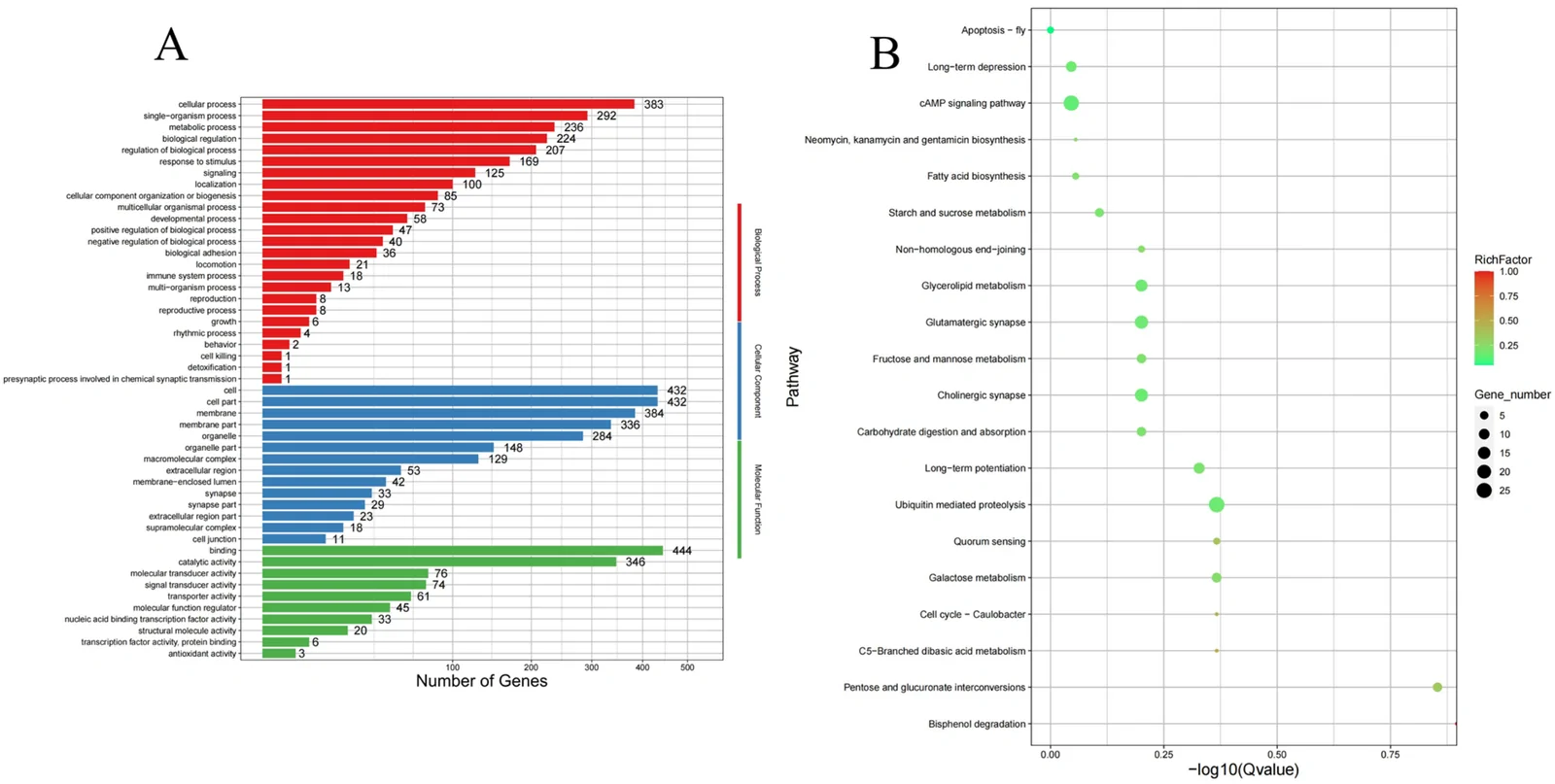
GO and KEGG enrichment analysis of differential TAD boundaries in the Q1 group. (**A**) Bar plot of GO enrichment for differential TAD boundaries in the Q1 group; (**B**) Bubble plot of KEGG enrichment for differential TAD boundaries in the Q1 group.

In the Q1 group, 250 of 3682 GO terms were significantly enriched (*p* < 0.05) (Figure 6A). Genes involved in biological processes (67.5%) were significantly enriched in cell–cell adhesion via plasma membrane adhesion molecules, positive regulation of catalytic activity, and positive regulation of hydrolase activity. Genes involved in cellular components (12.1%) were significantly enriched in synapse part, plasma membrane region, and postsynapse. Genes involved in molecular functions (20.3%) were significantly enriched in ionotropic glutamate receptor activity, glutamate receptor activity, and transmitter-gated ion channel activity. KEGG enrichment analysis of the Q1 group (Figure 6B) showed that a total of 234 KEGG pathways were enriched, among which 14 pathways were significantly enriched (*p* < 0.05), including bisphenol degradation, pentose and glucuronate interconversions, and cell cycle-related pathways (LONP2, peroxisomal protease). In summary, a portion of TADs (42.75%) were highly conserved stable genomic structural units across different genotypes of laying ducks, without changes in TAD boundaries, whereas those specific TAD boundaries, due to alterations, could cause changes in gene function (Table 3).

## 4. Discussion

Eggshell color, as a unique appearance trait of poultry eggs, has important economic value and has long attracted consumer attention. Blue-shelled eggs are favored by numerous consumers at home and abroad because of their unique eggshell color. In the duck egg market, green and white are two common eggshell colors. Studies have found that the formation of avian eggshell color is mainly determined by three pigments, namely protoporphyrin-IX, biliverdin-IX, and biliverdin zinc chelate [26]. Among them, the formation of blue-shelled eggs results from the deposition of biliverdin and its zinc chelate into the eggshell during egg formation [27]. It has been established that the site of eggshell formation is the uterus of the avian oviduct. Studies on biliverdin concentrations in the blood, eggshells, bile, and shell glands of brown-shelled and green-shelled laying hens found that the biliverdin concentration in the uterus of green-shelled hens was extremely significantly higher than that in brown-shelled hens, whereas no significant differences were found in blood or bile, suggesting that the secretion site of biliverdin is the uterus of the oviduct [28]. Based on the above physiological basis of the trait, this study focused on the shell glands of blue-shelled and white-shelled laying ducks to investigate the potential association between 3D chromatin architecture and eggshell color differentiation from a 3D genomic perspective.

The results of this study showed that both white-shelled laying ducks shell gland chromatin and blue-shelled laying ducks uterine chromatin exhibited extensive interactions across the entire genome. Compared with the white-shelled laying ducks group (B1), the blue-shelled laying ducks group (Q1) exhibited a relatively looser chromosomal structure (Figure 1). Studies show that relaxed chromatin conformation usually promotes transcription factor binding and regulatory element accessibility [29]. In this study, the relatively loose chromosomal structure in the shell glands of blue-shelled egg-laying ducks may create an open chromatin environment for transcription factors to recognize and bind regulatory regions of pigment deposition-related genes. Existing reports indicate that CTCF regulates global chromatin accessibility and transcriptional activity. CTCF binding sites overlap abundant promoters, and CTCF mainly enhances chromatin accessibility of its direct targets [30]. Our prior work demonstrated that the blue-shell allele had markedly higher CTCF binding affinity than the white-shell allele [31], implying that the blue-shell genotype could strengthen CTCF recruitment to sustain high chromatin accessibility in local and extended regions. Consequently, pigment deposition-related genes in the shell gland adopt a chromatin conformation conducive to transcription.

At the compartment level, intrachromosomal interactions in the duck shell gland exhibited a typical checkerboard distribution pattern (Figure 2A), and intrachromosomal interaction strength was significantly higher than interchromosomal interaction strength, which is consistent with observations in chickens [8] and mammals [3]. Higher-order chromatin can be divided into transcriptionally active A compartments, which are open chromatin regions containing active genes, and transcriptionally repressed B compartments [5]. This study found that the overall proportions of A/B compartments in the genomes of blue-shelled and white-shelled laying ducks were completely identical, with the proportion of compartment B (51.1%) slightly higher than that of compartment A (48.9%) (Figure 2B), suggesting that the difference in eggshell color phenotype did not cause global changes in the overall chromatin openness of duck shell gland and that the overall compartmental landscape was highly conserved between the two phenotypes. Meanwhile, the proportions of A/B compartments varied markedly among chromosomes (Figure 2C). Chromosome 31 was entirely an open A compartment in both groups, suggesting that the difference in eggshell color phenotype in laying ducks may be focused on chromatin conformational variation at local chromosomes or gene loci rather than global changes in the macroscopic A/B compartmental landscape across the genome. The GO and KEGG enrichment results of genes within differential compartments (Figure 3) showed that they were mainly involved in pathways such as immune response, basal metabolism, reproductive development, calcium signaling, and estrogen signaling, and no functional terms or regulatory pathways directly related to eggshell pigment synthesis, transport, or deposition were enriched, confirming that eggshell color differentiation in laying ducks does not depend on global or local structural variation of chromatin A/B compartments.

Studies have shown that TADs, as fine-scale chromatin topological regulatory units, can regulate gene interactions and transcriptional patterns within defined regions through boundary demarcation [32]. TADs are highly conserved across different species, cell types, and evolutionary stages [33]. Numerous studies have confirmed that non-coding genetic variations such as genomic deletions, inversions, and duplications can directly disrupt the integrity of TAD boundaries, causing local topological disturbances and ectopic rearrangement of distal regulatory elements, thereby breaking the original precise gene regulatory patterns and driving phenotypic variation in plants and animals [34,35,36]. This study found significant differences in TAD structure between the two groups of laying ducks, with obvious TAD boundary repositioning and loss on chromosome 4 in the blue-shelled laying ducks group (Q1) (Figure 4A). We analyzed the TAD structure of the shell glands from laying ducks with different eggshell colors at 20 kb resolution and found that the blue-shelled laying ducks group (Q1) had more TAD boundaries, shorter fragments, and significantly higher boundary signal strength (Figure 4B), with stable TADs as the predominant type, along with variation types such as merge, split, and rearrangement, indicating that specific local topological remodeling occurred in the shell glands of blue-shelled laying ducks. These findings suggest that eggshell phenotypic differences are associated with specific remodeling of local TAD topology.

In this study, 42.75% of TADs were found to be highly conserved between the two genotypes and were mainly responsible for maintaining genomic structural stability and basal physiological functions of the uterus, whereas the remaining specific TAD boundary variations may contribute to inter-group differences in molecular regulation. Functional annotation of genes at differential TAD boundaries further elucidated the biological effects of structural variation (Figure 5 and Figure 6). Genes at differential TAD boundaries in the white-shelled laying ducks group (B1) were mainly enriched in pathways such as nitrotoluene degradation, insect hormone biosynthesis, and limonene and pinene degradation; these genes also participated in pigment deposition-related pathways including porphyrin and chlorophyll metabolism and melanin biosynthesis (Table 3). Genes at differential TAD boundaries in the blue-shelled laying ducks group (Q1) were mainly enriched in pathways such as bisphenol degradation, pentose and glucuronate interconversions, and cell cycle—Caulobacter; these genes also participated in pigment deposition-related pathways including porphyrin and chlorophyll metabolism, riboflavin metabolism, bile secretion, and melanin biosynthesis (Table 3). Porphyrin metabolism serves as an essential upstream pathway for biliverdin biosynthesis, and biliverdin is the core pigment responsible for the blue eggshell in ducks [37]. We therefore hypothesize that the relocation and deletion of TAD boundaries on chromosome chr4 in blue-shelled ducks alter the spatial topological environment of local chromatin, regulate the expression of genes related to porphyrin metabolism, and ultimately participate in eggshell pigment deposition. Meanwhile, the differential pathways accompanied by TAD boundary changes suggest that, in addition to pigment synthesis pathways, pathways associated with cellular metabolism and substance transport are also co-regulated by chromatin topological remodeling.

This study has several important limitations. First, the sample size was limited (*n* = 2 biological replicates per group), which restricts statistical power and increases the potential influence of individual variation. Second, we did not perform functional validation to establish causal relationships between TAD remodeling and gene expression. Therefore, all findings presented here are preliminary and should be considered hypothesis generating rather than conclusive. Future studies with larger cohorts and functional assays are required to validate these observations.

## 5. Conclusions

In this study, Hi-C technology was used to perform a genome-wide analysis of the three-dimensional chromatin architecture in the shell glands of blue-shelled and white-shelled laying ducks, aiming to explore the epigenetic regulatory mechanism of the eggshell color trait. The results showed that the overall chromatin interaction patterns were conserved between blue-shelled and white-shelled laying ducks, but their spatial folding patterns differed significantly. Blue-shelled laying ducks preferred short-range loose interactions, whereas white-shelled laying ducks exhibited predominantly medium- and long-range looping interactions. The A/B compartment structures were highly stable between the two groups, and genes in differential compartments were not enriched in pigment deposition-related pathways, indicating that eggshell color variation did not depend on macroscopic chromatin compartment remodeling. In contrast, chromosome 4 in blue-shelled laying ducks underwent specific TAD boundary remodeling, which was associated with the differential expression of genes in pigment deposition pathways. In summary, local TAD topological variation may serve as a potential three-dimensional genomic regulatory basis for eggshell color differentiation. However, given the limited sample size and lack of functional validation, these findings are preliminary and require further verification.

## Figures and Tables

**Table 1:** Overview of Hi-C sequencing data.

Sample	Clean Reads	Base Count (bp)	Read Length (bp)	Q20%	Q30%	GC Content %	Error Rate %
B1_1	895631558	267395084582	148.5	98.1	93.8	42.6	0.23
B1_2	892214818	266456622616	149.0	98.2	93.9	42.4	0.25
Q1_1	880316491	262860568304	149.0	98.2	93.9	43.3	0.27
Q1_2	869407040	259655139864	149.0	98.3	94.1	43.4	0.27

**Table 2:** Proportions of intrachromosomal and interchromosomal interactions.

Sample	Mapped Reads	Valid Pairs	Cis Valid Pairs	Trans Valid Pairs	Cis/Trans
B1_1	663,587,096 (100%)	432,571,478 (65.19%)	314,715,237 (72.75%)	117,856,241 (27.25%)	2.67
B1_2	675,390,684 (100%)	417,976,137 (61.89%)	302,777,938 (72.44%)	115,198,199 (27.56%)	2.63
Q1_1	635,676,020 (100%)	372,648,108 (58.62%)	287,597,308 (77.18%)	85,050,800 (22.82%)	3.38
Q1_2	627,447,597 (100%)	370,284,227 (59.01%)	284,664,408 (76.88%)	85,619,819 (23.12%)	3.32

**Table 3:** Genes related to pigment deposition and KEGG pathway.

Item	KEGGE Pathway	*p*-Value	KEGG ID
B1	Porphyrin and chlorophyll metabolism	0.452	ko00860
	Melanogenesis	0.818	ko04916
Q1	Phenylalanine metabolism	0.479	ko00860
	Riboflavin metabolism	0.178	ko00740
	Bile secretion	0.594	ko04976
	Melanogenesis	0.753	ko04916

## Data Availability

The raw data supporting the conclusions of this article will be made available by the authors on request. The Hi-C sequencing data are currently being prepared for submission to a public repository (NCBI GEO/SRA, https://www.ncbi.nlm.nih.gov/geo/, accessed on 26 October 2026) and will be made publicly available upon acceptance of the manuscript.

## Supplementary Materials

The following supporting information can be downloaded at https://www.mdpi.com/article/10.3390/biology15211957/s1, Table S1: Number and length of AB compartments in B1 and Q1; Table S2: GO annotation results of genes within differential TAD boundaries in the B1 group; Table S3: KEGG annotation results of genes within differential TAD boundaries in the B1 group; Table S4: GO annotation results of genes within differential TAD boundaries in the Q1 group; Table S5: KEGG annotation results of genes within differential TAD boundaries in the Q1 group.

## Abbreviations

The following abbreviations are used in this manuscript:

Hi-C	High-throughput chromosome conformation capture
3D	Three-dimensional
Q1	Blue-shelled laying ducks
B1	White-shelled laying ducks
TADs	Topologically associating domains

## References

[1] Haws S.A., Simandi Z., Barnett R.J., Phillips-Cremins J.E. (2022). 3D genome, on repeat: Higher-order folding principles of the heterochromatinized repetitive genome. Cell.

[2] Bickmore W.A. (2013). The spatial organization of the human genome. Annu. Rev. Genom. Hum. Genet..

[3] Zheng Y., Zhang L., Jin L., Zhang P., Li F., Guo M., Gao Q., Zeng Y., Li M., Zeng W. (2022). Unraveling three-dimensional chromatin structural dynamics during spermatogonial differentiation. J. Biol. Chem..

[4] Lupiáñez D.G., Kraft K., Heinrich V., Krawitz P., Brancati F., Klopocki E., Horn D., Kayserili H., Opitz J.M., Laxova R. (2015). Disruptions of topological chromatin domains cause pathogenic rewiring of gene-enhancer interactions. Cell.

[5] Lieberman-Aiden E., van Berkum N.L., Williams L., Imakaev M., Ragoczy T., Telling A., Amit I., Lajoie B.R., Sabo P.J., Dorschner M.O. (2009). Comprehensive mapping of long-range interactions reveals folding principles of the human genome. Science.

[6] Shao D., Yang Y., Shi S., Tong H. (2022). Three-Dimensional Organization of Chicken Genome Provides Insights into Genetic Adaptation to Extreme Environments. Genes.

[7] Fishman V., Battulin N., Nuriddinov M., Maslova A., Zlotina A., Strunov A., Chervyakova D., Korablev A., Serov O., Krasikova A. (2019). 3D organization of chicken genome demonstrates evolutionary conservation of topologically associated domains and highlights unique architecture of erythrocytes’ chromatin. Nucleic Acids Res..

[8] Maslova A., Plotnikov V., Nuriddinov M., Gridina M., Fishman V., Krasikova A. (2023). Hi-C analysis of genomic contacts revealed karyotype abnormalities in chicken HD3 cell line. BMC Genom..

[9] Scanes C.G., WHITTOWGC (2021). Reproduction in the female. Sturkie’s Avian Physiology.

[10] Zhang F., Yin Z.T., Zhang J.F., Zhu F., Hincke M., Yang N., Hou Z.C. (2020). Integrating transcriptome, proteome and QTL data to discover functionally important genes for duck eggshell and albumen formation. Genomics.

[11] Altmann B.A., Trinks A., Mörlein D. (2023). Consumer preferences for the color of unprocessed animal foods. J. Food Sci..

[12] Cheng X., Ning Z. (2023). Research progress on bird eggshell quality defects: A review. Poult. Sci..

[13] Zhang X., Li Y., Chen J. (2026). Eggshell Biomineralization and Genetic Regulation of Eggshell Strength of Laying Hens. Agriculture.

[14] Gautron J., Stapane L., Le Roy N., Nys Y., Rodriguez-Navarro A.B., Hincke M.T. (2021). Avian eggshell biomineralization: An update on its structure, mineralogy and protein tool kit. BMC Mol. Cell Biol..

[15] Stapane L., Le Roy N., Ezagal J., Rodriguez-Navarro A.B., Labas V., Combes-Soia L., Hincke M.T., Gautron J. (2020). Avian eggshell formation reveals a new paradigm for vertebrate mineralization via vesicular amorphous calcium carbonate. J. Biol. Chem..

[16] Sun T., Xiao C., Yang Z., Deng J., Yang X. (2023). Transcriptome profiling analysis of uterus during chicken laying periods. BMC Genom..

[17] Bolger A.M., Lohse M., Usadel B. (2014). Trimmomatic: A flexible trimmer for Illumina sequence data. Bioinformatics.

[18] Wingett S.W., Andrews S. (2018). FastQ Screen: A tool for multi-genome mapping and quality control. F1000Research.

[19] Ay F., Bailey T.L., Noble W.S. (2014). Statistical confidence estimation for Hi-C data reveals regulatory chromatin contacts. Genome Res..

[20] Crane E., Bian Q., McCord R.P., Lajoie B.R., Wheeler B.S., Ralston E.J., Uzawa S., Dekker J., Meyer B.J. (2015). Condensin-driven remodelling of X chromosome topology during dosage compensation. Nature.

[21] Zheng X., Zheng Y. (2018). CscoreTool: Fast Hi-C compartment analysis at high resolution. Bioinformatics.

[22] Ke Y., Xu Y., Chen X., Feng S., Liu Z., Sun Y., Yao X., Li F., Zhu W., Gao L. (2017). 3D Chromatin Structures of Mature Gametes and Structural Reprogramming during Mammalian Embryogenesis. Cell.

[23] Xie C., Mao X., Huang J., Ding Y., Wu J., Dong S., Kong L., Gao G., Li C.Y., Wei L. (2011). KOBAS 2.0: A web server for annotation and identification of enriched pathways and diseases. Nucleic Acids Res..

[24] Kanehisa M., Goto S. (2000). KEGG: Kyoto encyclopedia of genes and genomes. Nucleic Acids Res..

[25] Young M.D., Wakefield M.J., Smyth G.K., Oshlack A. (2010). Gene ontology analysis for RNA-seq: Accounting for selection bias. Genome Biol..

[26] Hamchand R., Hanley D., Prum R.O., Brückner C. (2020). Expanding the eggshell colour gamut: Uroerythrin and bilirubin from tinamou (Tinamidae) eggshells. Sci. Rep..

[27] Hanley D., Grim T., Cassey P., Hauber M.E. (2015). Not so colourful after all: Eggshell pigments constrain avian eggshell colour space. Biol. Lett..

[28] Zhao R., Xu G.Y., Liu Z.Z., Li J.Y., Yang N. (2006). A study on eggshell pigmentation: Biliverdin in blue-shelled chickens. Poult. Sci..

[29] Senapati S., Irshad I.U., Sharma A.K., Kumar H. (2025). Predicting gene expression changes from chromatin structure modification. npj Syst. Biol. Appl..

[30] Chen D., Keremane S., Wang S., Lei E.P. (2025). CTCF regulates global chromatin accessibility and transcription during rod photoreceptor development. Proc. Natl. Acad. Sci. USA.

[31] Chen L., Gu X., Huang X., Liu R., Li J., Hu Y., Li G., Zeng T., Tian Y., Hu X. (2020). Two cis-regulatory SNPs upstream of ABCG2 synergistically cause the blue eggshell phenotype in the duck. PLoS Genet..

[32] Szabo Q., Bantignies F., Cavalli G. (2019). Principles of genome folding into topologically associating domains. Sci. Adv..

[33] Dixon J.R., Gorkin D.U., Ren B. (2016). Chromatin Domains: The Unit of Chromosome Organization. Mol. Cell.

[34] Kabirova E., Ryzhkova A., Lukyanchikova V., Khabarova A., Korablev A., Shnaider T., Nuriddinov M., Belokopytova P., Smirnov A., Khotskin N.V. (2024). TAD border deletion at the Kit locus causes tissue-specific ectopic activation of a neighboring gene. Nat. Commun..

[35] Wang Z., Tian W., Guo Y., Wang D., Zhang Y., Zhi Y., Li D., Li W., Li Z., Jiang R. (2024). Dynamic alternations of three-dimensional chromatin architecture contribute to phenotypic characteristics of breast muscle in chicken. Commun. Biol..

[36] Salnikov P., Korablev A., Serova I., Belokopytova P., Yan A., Stepanchuk Y., Tikhomirov S., Fishman V. (2024). Structural variants in the Epb41l4a locus: TAD disruption and Nrep gene misregulation as hypothetical drivers of neurodevelopmental outcomes. Sci. Rep..

[37] Wang L., Yu S., Zhang Y., Guo Z., Wang S., Hou S. (2025). Integrated transcriptomic and metabolomic analysis reveals mechanisms of eggshell color variation in Pekin ducks. Poult. Sci..

